# A Conceptual Playground Between Perception and Cognition: Introduction to the Special Issue on Amodal Completion

**DOI:** 10.1177/2041669520939108

**Published:** 2020-07-06

**Authors:** Rob van Lier, Vebjørn Ekroll

**Affiliations:** Donders Institute for Brain, Cognition and Behaviour, Radboud University, Nijmegen, The Netherlands; Department of Psychosocial Science, University of Bergen, Bergen, Norway

In the past decades, the notion of *amodal completion* has developed into a well-known concept in perception theory. The enigmatic completion phenomena raise fundamental theoretical questions about the nature of perception and cognition, and although significant progress has been made, they remain a conceptual challenge for perception theory in general. We have only begun to explore the rich conceptual playground it provides.

The term *amodal completion* is widely used in the literature, but is also rather puzzling. What does it mean for a perceptual interpretation to be amodal—not constrained to the given modality of perception? Phenomenologically, the adjective *amodal* refers to the fact we actually do not *see* the partly occluding parts of an object or scenery. The appearance of any distal stimulus obviously belongs to the outcome of the perceptual process, revealing sensory qualities for some parts, but lacking for other parts—while still being inextricable blended with the overall percept. However, regarding the underlying processes themselves, the term *amodal* appears less clear-cut. Note that the “complements amodeaux” in the original French title of Michotte’s et al. (1964) famous booklet indeed referred to the result of the completion process, not so much the process itself (e.g., Van Lier & Gerbino, 2015).

Ever since the pioneering work of Burke, Michotte, and Kanizsa (Burke, 1952; Kanizsa, 1979; Michotte & Burke, 1951; Michotte et al., 1964), the phenomenon of amodal completion has posed intriguing opportunities and challenges for cognitive science. Because the perception of occluded scene regions involves quite an extreme level of stimulus incompleteness and ambiguity, it appears particularly well-suited for studying the rules and principles of perception. As Gerbino (2020) highlights in his historical review, paraphrasing Koffka’s (1935) original ideas, “perceptual completions (both modal and amodal) are key phenomena because they reveal inner forces of organization, when outer forces are weak or absent.” Perhaps most importantly, amodal completion challenges our naïve ideas about the distinction between seeing and thinking. Since amodal completion refers to mental experiences of occluded, and hence invisible regions in a visible scene, it appears odd to conceptualize it as seeing, yet these mental experiences tend to behave like visual impressions in many ways. They often force themselves upon the viewer in an automatic and strangely compelling way, and like most visual illusions, they tend to persist even when they are in direct conflict with the viewer’s conscious knowledge.

Interestingly, among the most ardent proponents of a strict dichotomy between perception and cognition, we find the major pioneers of research on amodal completion, as well as other major theorists who marshal observations from research on amodal completion as arguments for their position. For example, referring to Kanizsa’s (1985) classic work, Pylyshyn (1999) notes that particularly “revealing examples of the difference between the organizing principles of vision and the principles of inference are to be found in the phenomenon of ‘amodal completion’” (p. 344). Similarly, Firestone and Scholl (2015) note that perhaps “nobody has elucidated the empirical foundations and theoretical consequences of this observation [that what you see can be different from what you think] better than Gaetano Kanizsa, whose ingenious demonstrations of such conflict can, in a single figure, obliterate the worry that perception and cognition are merely ‘folk categories’ […]” (p. 54).

Because amodal completion challenges our ideas about what it means to see, we believe that amodal completion is of pivotal importance for theories of perception. We also believe that it offers a particularly rich conceptual playground at the demarcation line (or, alternatively, in the gray zone) between perception and cognition. A main reason why we launched a call for contributions to a special issue on amodal completion was that we felt that—although much progress has been made over the last 50 years—amodal completion is currently not being studied as vigorously as we would expect based on our belief that it is of central theoretical importance to perception theory. Thus, as we launched the call, it was not without a fair amount of worry that we might not be able to attract enough submissions for a decent special issue. As the submissions started to come in, however, we were delighted to see that our worries were unfounded.

In light of the highly diverse contributions to this special issue, it is evident that amodal completion is much more than just a curious oddity. Rather than being a limited topic of purely academic interest, amodal completion appears to play a pervasive role in our lives. The present special issue provides a wealth of beautiful phenomena and experimental contributions involving amodal completion, using abstract stimuli to test Gestalt-like processing, both in static displays (Chen et al., 2018; Peta et al., 2019) and dynamic displays (Anstis, 2018; Nakajima et al., 2019; Tyler, 2019), or more complex stimuli, for example, using stereoscopic fusion (Tse, 2017a, 2017b) or human faces (Haberman & Ulrich, 2019), whereas other contributions highlight completion phenomena in a broad range of domains like art and design (Koenderink et al., 2018; van Lier & Ekroll, 2019), magic (Ekroll, De Bruyckere, et al., 2018), architecture (Ekroll, Mertens, et al., 2018), fashion (Kiritani et al., 2018), and even the history of astronomy (Roncato, 2019). Besides that, this special issue also comprises extensive conceptual reviews from different angles: perceptual psychology (Gerbino, 2020; Scherzer & Faul, 2019), philosophy (Nanay, 2018), and neurosciences (Thielen et al., 2019).

Critical notes and thoughtful insights on the nature of amodal completion are scattered throughout the aforementioned contributions, focusing on issues such as the distinction and commonalities between modal and amodal completion (e.g., Koenderink et al., 2018; Scherzer & Faul, 2019; Tse, 2017a, 2017b), the perception-cognition dichotomy (e.g., Ekroll, Mertens, et al., 2018; Gerbino, 2020; Nanay, 2018; Roncato, 2019; van Lier & Ekroll, 2019), or the nature of the underlying representations (e.g., Gerbino, 2020; Haberman & Ulrich, 2019; Koenderink et al., 2018; Scherzer & Faul, 2019; Thielen et al., 2019). One issue stands out when considering the diversity of the present collection of papers. Although the term amodal completion suggests that we are dealing with a single unified concept, the underlying processes are probably multiple (see also, Gerbino, 2020; Thielen et al., 2019). Amodal presence might very well be as rich as modal presence when it comes to determining underlying processing aspects. We trust and hope that the present collection helps in putting amodal completion at the forefront of perception research. It is our belief that grasping the nature of amodal completion brings us further into unraveling the true nature of perception.
